# *TXNDC2* joint molecular marker is associated with testis pathology and is an accurate predictor of sperm retrieval

**DOI:** 10.1038/s41598-021-92603-3

**Published:** 2021-06-22

**Authors:** Seyed-Morteza Javadirad, Mohammad Mokhtari

**Affiliations:** 1 Department of Cell and Molecular Biology and Microbiology, Faculty of Biological Science and Technology, University of Isfahan, 81746-73441 Isfahan, Iran; 2 Department of Molecular Genetics, Faculty of Biological Sciences, Tarbiat Modares University, 14115-111 Tehran, Iran

**Keywords:** Predictive markers, Testis, Genetic markers

## Abstract

The association of *PRM1*/*2* with male azoospermia is well-documented, but the relationship between *TXNDC2* deficiency and the azoospermia phenotype, sperm retrieval, and pathology has not been elucidated. Here we identified the association of *TXNDC2* and protamines in evaluating testis pathology and sperm retrieval. An extensive microarray meta-analysis of men with idiopathic azoospermia was performed, and after undergoing several steps of data quality controls, the data passing QC were pooled and batch effect corrected. As redox imbalance has been shown to have a variable relationship with fertility, our relative expression studies began with candidate protamination and thioredoxin genes. We constructed a logistic regression model of *TXNDC2* with *PRM1* and *PRM2* genes, and collective ROC analysis indicated a sensitivity of 96.8% and specificity of 95.5% with a ROC value of 0.995 (SE = 0.0070, 95% CI 0.982–1.000). These results demonstrate that *TXNDC2*, *PRM1*, and *PRM2* combined have a robust power to predict sperm retrieval and correlate with severe azoospermia pathology.

## Introduction

Obstructive (OA) and non-obstructive azoospermia (NOA) denote normal and abnormal spermatogenesis, respectively. Aberrant spermatogenesis is also classified into five main pathologic patterns^1^: seminiferous tubule hyalinization (SH), Sertoli cell-only syndrome (SCOS), early maturation arrest (eMA), late maturation arrest (lMA), and hypospermatogenesis (Hypo). Ultimately, pathological analyses can identify spermatogenesis failure and ductal obstruction; however, sperm retrieval (SR) cannot be predicted solely based on the current approach.

Medical expenses and loss of golden time are two factors preventing the treatment of azoospermic men wishing to have biological children. Reliable and precise molecular markers, especially those detecting spermatogenesis pathology, could be a boon for would-be parents. To reduce infertility stress on couples and improve male fertility, especially for NOA men, we previously introduced the *KDM3A* to *PRM1* expression ratio as a reliable molecular indicator of SR^2^. However, we have thus far not been able to detect any association between the aforementioned genes and the pathological features of the biopsies. It is critical to identify the gene(s) that will allow us to predict the success of SR while confirming testicular pathology. By joining pathology and genetics in this manner, we can determine the possibility of SR. This information could persuade surgeons to explore tissues from NOA men to extract any residual sperm during the first micro-TESE surgery.

Thioredoxins are intracellular and extracellular scavengers of the oxidative stress system. Reactive oxygen species (ROS) are one of their main targets, and the regulation of redox signaling plays pivotal roles in sperm fertility^3^. Thioredoxin domain-containing 2 (*TXNDC2*, ENSG00000168454) is transiently expressed in the haploid phase of spermatogenesis and, as a sperm-specific oxidoreductase, is only detected in round and elongating spermatids^4,5^. Double inactivation of *TXNDC2*/*TXNDC3* was performed in animal models, and the output was impaired chromatin protamination^6^. A DNA safeguard, protamination not only condensates sperm chromatin but also replaces most histones during spermiogenesis; male infertility is conclusively associated with impaired protamination^7^. Known to begin with the expression of transition protein 1 (*TNP1*), protamination is followed by protamine (*PRM1* and *PRM2*) replacement in the nucleus^8^. Thereafter, mature spermatozoa are released into the lumen of seminiferous tubules^9^, and capacitation then starts as the final step of sperm maturation. Even after capacitation, decondensation of sperm chromatin would be triggered by heparin sulfate of mammalian oocytes^10^, a phenomenon highlighting how previous chromatin condensation is necessary for male fertility.

In this study, *TXNDC3* was not evaluated as it is ubiquitously expressed in all tissues and is no longer considered testis specific^11^. Considering *TXNDC2* is localized in the nucleus and *TXNDC8* is distributed extracellularly, the latter was also removed from analyses. Therefore, the aim of this study was to evaluate the expression levels of *TXNDC2* concomitantly with protamination genes in different azoospermia pathologies. We showed that *PRM1* and *PRM2*, but not *TNP1*, are excellent indicators of SR. We also showed that *TXNDC2* expression levels were consistent with tissue pathologies. Moreover, logistic regression model analysis of combined *TXNDC2*, *PRM1*, and *PRM2* genes was a robust predictor of SR, providing a sensitivity of 96.8% and specificity of 95.5%.

## Results

### Data quality control and pre-processing

The assessment of data normalization revealed that parts of the data were log_2_ scaled, and the remainder were transformed. The second round of quality control was carried out to assess the quality of sample quantiles (Supplementary Fig. 1). For each dataset, hierarchical cluster analysis of samples, based on Euclidian Distance of the Pearson correlation coefficient, grouped similar objects into clusters. Clustering was followed by dimension reduction using the Eigenvector with the highest Eigenvalue (Supplementary Fig. 2). The decision to remove 27 outliers out of 89 samples was based on advanced knowledge of biology, combined with clustering and PCA (supplementary Fig. 3). Consequently, a total of 62 samples were pooled for further analyses.

**Figure 1:**
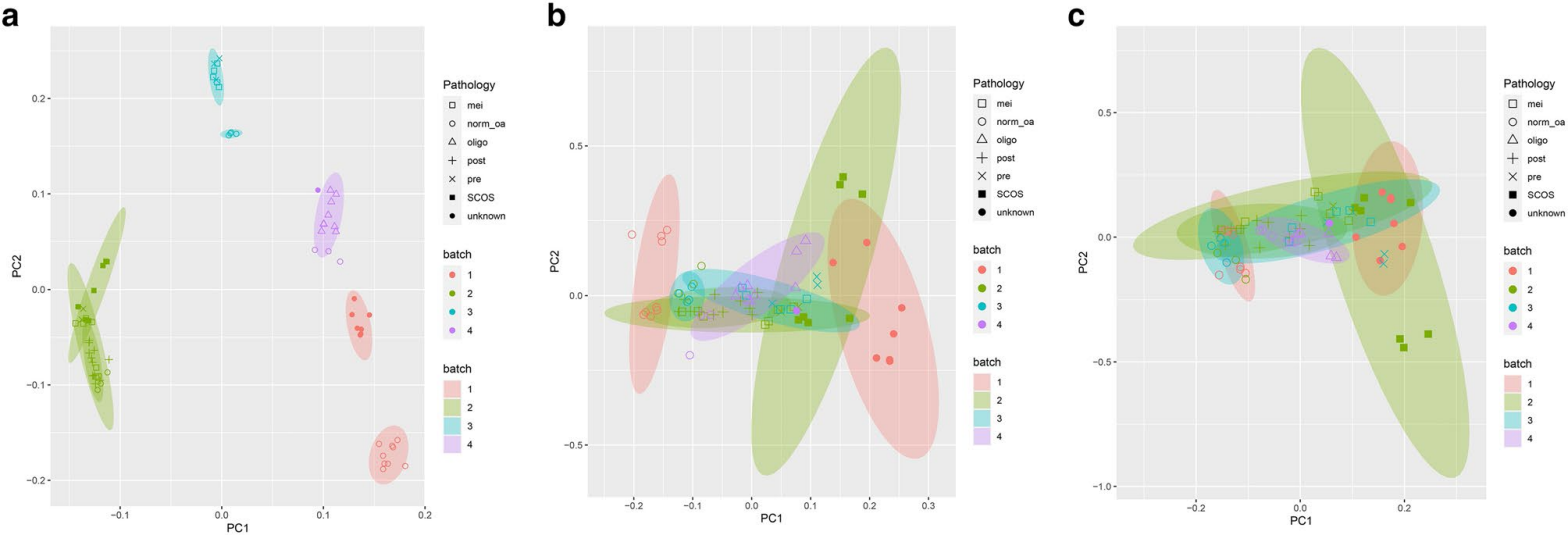
PCA of pooled samples before and after the batch effect removal (using different algorithms). (**a**) Before the batch effect removal, samples with identical or similar pathology were separated based on their batches. After the removal, PCA separated samples according to their pathology and the samples were grouped regardless of their batches using limma algorithm (**b**) and SVA algorithm (**c**). Batch 1–4 represents GSE145467, GSE45885, GSE108886, GSE14310. mei (meiotic arrest); norm_oa (normal spermatogenesis or obstructive azoospermia); oligo (oligospermia); post (post meiotic arrest); pre (pre meiotic arrest); SCOS (Sertoli cell-only syndrome); unknown (azoospermia with unknown pathology).

Limma and SVA algorithms were applied to the pool to correct their batch. Hierarchical clustering and PCA were performed, and the outcome provided the confidence about the correction (Fig. 1).

### Meta-analysis

**Figure 2:**
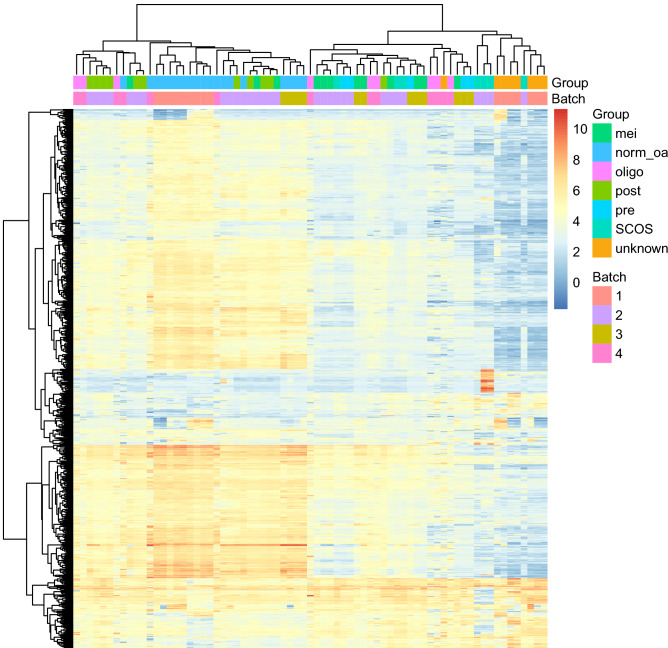
A heatmap representing 71 samples, clustered based on correlation coefficient of 788 genes with standard deviation greater than 1. Group indicates the pathology of samples and the batch represents different datasets. Batch effect removal was approved as the heatmap clusters genes based on their pathologic groups and separates them based on their batches. Batch 1–4 represents GSE145467, GSE45885, GSE108886, GSE14310. mei (meiotic arrest); norm_oa (normal spermatogenesis or obstructive azoospermia); oligo (oligospermia); post (post meiotic arrest); pre (pre meiotic arrest); SCOS (Sertoli cellonly syndrome); unknown (azoospermia with unknown pathology).

**Figure 3:**
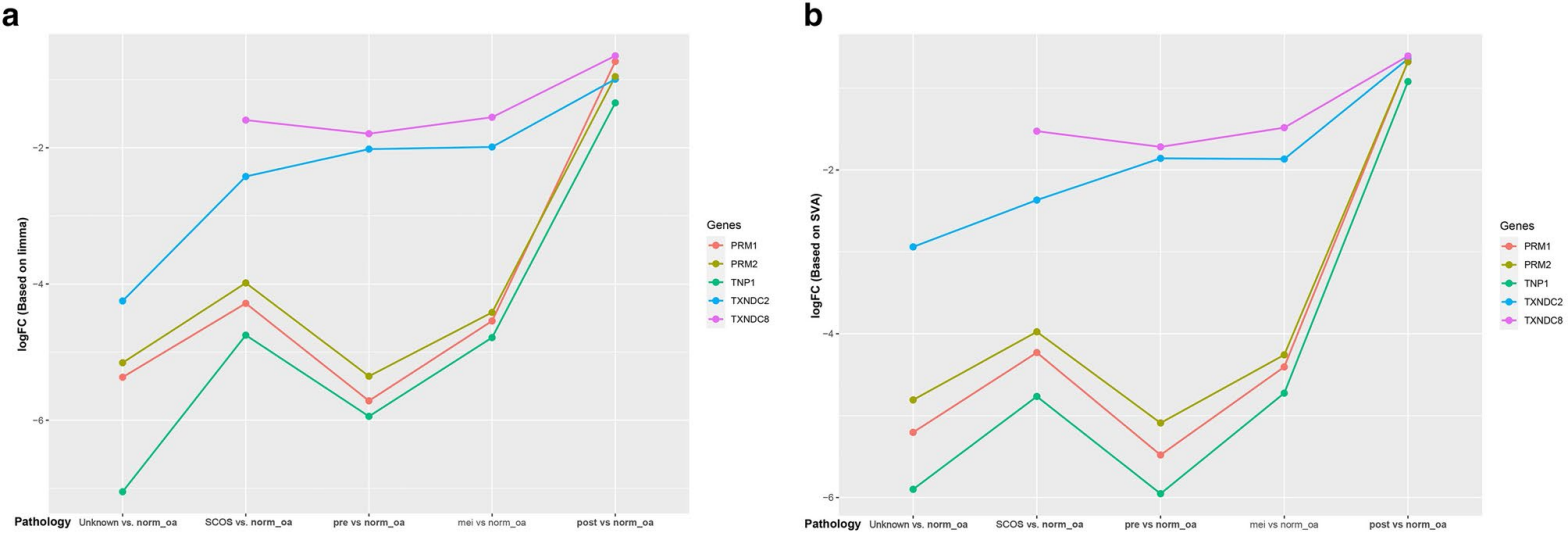
Log fold changes of TXNDC2, PRM1, PRM2 and TNP1 genes in different pathologies were illustrated. (**a**) After the batch effect removal using limma package, different log2FC of individual genes was visualized in different aberrant pathologies. (**b**) A same pattern of log2FC differences were also observed after batch effect removal, using SVA algorithm. In all comparisons, normal spermatogenesis was used as control. mei (meiotic arrest); norm_oa (normal spermatogenesis or obstructive azoospermia); oligo (oligospermia); post (post meiotic arrest); pre (pre meiotic arrest); SCOS (Sertoli cell-only syndrome); unknown (azoospermia with unknown pathology); TXNDC2 (Thioredoxin Domain Containing 2); TXNDC8 (Thioredoxin Domain Containing 8); PRM1 (Protamine 1); PRM2 (Protamine 2); TNP1 (Transition Protein 1).

The gene expression of pooled data with pathological phenotypes of SCOS (7 samples), pre-meiotic arrest (5 samples), meiotic arrest (12 samples), and post-meiotic arrest (11 samples) was evaluated (Fig. 2). Based on the goal of this study, protamination genes (*PRM1*, *PRM2*, *TNP1*) with respect to testis-specific thioredoxin genes (*TXNDC2*, *TXNDC8*) were analyzed (Table 1 and Fig. 3). SCOS patients’ meta-analysis revealed meaningful downregulation of *TXNDC2* (effect size = − 2.42, FDR = 7.86E−07), *PRM1* (effect size = − 4.28, FDR = 5.89E−07), *PRM2* (effect size = − 3.98, FDR = 1.77E−06), and *TNP1* (effect size = − 4.75, FDR = 8.32E−09). Similar meaningful downregulation of the genes was also recorded in pre-meiotic arrest and meiotic arrest phenotypes, but not in post-meiotic arrest. *TXNDC2* (effect size = − 4.25, FDR = 1.44E−15), *PRM1* (effect size = − 5.37, FDR = 1.99E−10), *PRM2* (effect size = − 5.16, FDR = 3.60E−10), and *TNP1* (effect size = − 7.05, FDR = 6.48E−16) were all downregulated in the idiopathic azoospermia dataset. Except for post-meiotic arrest, *TXNDC8* meaningful downregulation was detected for SCOS (effect size = − 1.59, FDR = 3.97E−05), pre-meiotic arrest (effect size = − 1.79, FDR = 8.63E−05) and meiotic arrest (effect size = − 1.55, FDR = 4.53E−05).

**Table 1:** GEO analysis results.

GEO accession number (number of samples)	Pathology (number of samples)	Gene name	SVA		Limma	
			Effect size	FDR	Effect size	FDR
GSE145467 (16) GSE45885 (30) GSE108886 (12) GSE14310 (4)	Unknown vs. norm_oa (8 vs. 19)	TXNDC2	− 2.93896	1.14E−09	− 4.24932	1.44E−15
		PRM1	− 5.20465	1.91E−10	− 5.36864	1.99E−10
		PRM2	− 4.80788	2.12E−09	− 5.15656	3.60E−10
		TNP1	− 5.89948	1.09E−11	− 7.0504	6.48E−16
	SCOS vs. norm_oa (7 vs. 19)	TXNDC2	− 2.36736	7.16E−07	− 2.42145	7.86E−07
		PRM1	− 4.22982	1.59E−07	− 4.28438	5.89E−07
		PRM2	− 3.97574	6.48E−07	− 3.98456	1.77E−06
		TNP1	− 4.76556	1.86E−08	− 4.75112	8.32E−09
	Pre vs. norm_oa (5 vs. 19)	TXNDC2	− 1.85791	0.000951	− 2.01963	0.000557
		PRM1	− 5.48043	3.84E−08	− 5.71566	6.07E−08
		PRM2	− 5.08952	2.22E−07	− 5.35397	1.81E−07
		TNP1	− 5.95316	1.25E−08	− 5.94338	4.08E−09
	Mei vs. norm_oa (12 vs. 19)	TXNDC2	− 1.8668	1.12E−05	− 1.98834	3.97E−06
		PRM1	− 4.40475	3.15E−09	− 4.54281	5.81E−09
		PRM2	− 4.25746	8.18E−09	− 4.41748	7.07E−09
		TNP1	− 4.72509	1.13E−09	− 4.78687	1.50E−10
	Post vs. norm_oa (11 vs. 19)	TXNDC2	− 0.64269	0.482129	− 0.99323	0.113607
		PRM1	− 0.67478	0.727302	− 0.73242	0.669765
		PRM2	− 0.67873	0.725413	− 0.95463	0.506082
		TNP1	− 0.9204	0.602481	− 1.3407	0.227524
	SCOS vs. norm_oa (7 vs. 7)	TXNDC8	− 1.52593	4.46E−05	− 1.59405	3.97E−05
	Pre vs. norm_oa (5 vs. 7)		− 1.71766	8.23E−05	− 1.79352	8.63E−05
	Mei vs. norm_oa (12 vs. 7)		− 1.48322	4.45E−05	− 1.55205	4.53E−05
	Post vs. norm_oa (11 vs. 7)		− 0.60822	0.2240309	− 0.65034	0.21628

GSE145467, GSE45885, GSE108886 and GSE14310 were analyzed. Normal spermatogenesis was compared with different pathologies of azoospermia including SCOS and meiotic arrests. Gene names were according to Hugo nomenclature outline. Expression fold changes are Log2 scaled (Log2FC) according to the limma and SVA packages. Absolute FC was calculated based on Log2FC. False discovery rate (FDR) is Benjamini Hochberg correction of the p-values. Pathology represents mei: meiotic arrest, norm_oa: normal spermatogenesis or obstructive azoospermia, oligo: oligospermia, post: post meiotic arrest, pre: pre meiotic arrest, SCOS: Sertoli cell-only syndrome, unknown: azoospermia with unknown pathology. Gene name represents TXNDC2: Thioredoxin Domain Containing 2, TXNDC8: Thioredoxin Domain Containing 8, PRM1: Protamine 1, PRM2: Protamine 2, TNP1: Transition Protein 1s.

### RT-qPCR data analysis

**Figure 4:**
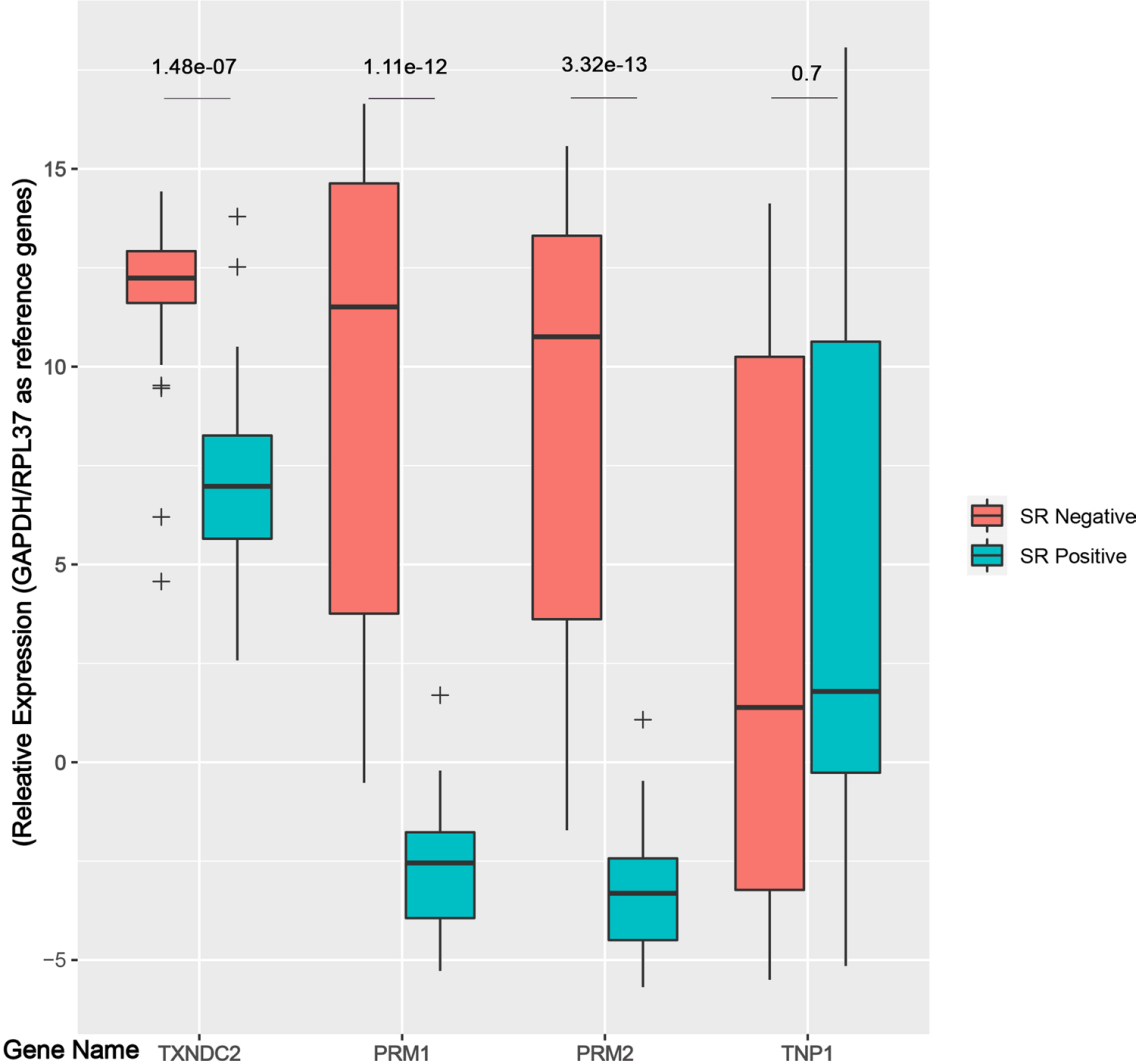
Relative expression of TXNDC2 and protamination genes were compared between men with positive (blue bars) and negative (red bars) sperm retrieval. Mean Cqs of both reference genes, GAPDH and RPL37, were calculated and used for relative expression. Meaningful intra-gene differences were illustrated for TXNDC2, PRM1 and PRM2. TNP1 showed overlapped relative expression between samples with positive and negative sperm retrieval. p-value less than 0.05 were considered as significant.

The mean expression level of *GAPDH*, *RPL37*, *TXNDC2*, *PRM1*, *PRM2*, and *TNP1* was compared between positive and negative SR (Supplementary Table 1). Reference genes *GAPDH* and *RPL37* showed the minimal mean differences between positive and negative SR individuals (0.59 and 0.97, respectively). High positive mean differences were detected for *TXNDC2*, *PRM1*, and *PRM2* (considering positive SR as the control). However, *TNP1* showed a negative (− 1.52) mean difference. Therefore, *TXNDC2* was differentially expressed in homology and protamination genes *PRM1* and *PRM2*. Unexpectedly, the expression of *TNP1* was overlapping (Fig. 4). A t-test was performed on normalized data to determine the significance of the observed differences (Table 2). A significant differential expression for *TXNDC2*, *PRM1*, and *PRM2* (p = 0.000) was observed between positive and negative SR, but not for *TNP1* (p = 0.558).

**Table 2:** Sperm retrieval and mRNA expression.

Sperm retrieval status	Number (percent)	Mean differences (p value)					
		GAPDH^b^	RPL37^a^	TXNDC2^b^	PRM1^a^	PRM2^a^	TNP1^a^
Positive	23 (40.351)	− 0.906 (0.063)	− 0.016 (0.105)	− 5.724 (0.000)	− 0.255 (0.000)	− 0.263 (0.000)	− 0.227 (0.558)
Negative	34 (59.649)						
Total	57 (100)						

t-Test has been used to compare the mean mRNA expression of GAPDH, RPL37, TXNDC2, PRM1, PRM2 and TNP1 between men with different sperm retrieval status.

^a^Log10 transformation was used for normalization and the normalized data was used for analysis.

^b^Raw data was normally distributed and therefore it was used for analysis.

REST2009 relative expression analysis results are presented in Table 3. Data analysis showed significant downregulation of *TXNDC2* with an expression ratio of 0.047 (p = 0.000). *PRM1* and *PRM2* genes were also significantly (p = 0.000) downregulated with an expression ratio of 0.000. *TNP1*, on the other hand, was insignificantly (p = 0.301) upregulated with a minor expression ratio of 4.078.

**Table 3:** Relative expression report deduced from REST2009.

Gene	Type	Reaction efficiency	Expression	Std. error	95% C.I.	P(H1)	Result
GAPDH	REF	1.00	1.276				
RPL37	REF	0.92	0.783				
TXNDC2	TRG	1.00	0.047	0.005–0.559	0.001–10.258	0.000	DOWN
PRM1	TRG	1.00	0.000	0.000–0.036	0.000–0.688	0.000	DOWN
PRM2	TRG	0.93	0.000	0.000–0.041	0.000–0.834	0.000	DOWN
TNP1	TRG	0.90	4.078	0.002–4750.851	0.000–1,237,021.157	0.301	

SFA individuals were compared to SRA and two reference genes, GAPDH and RPL37, were applied simultaneously for quantification. P(H1)—Probability of alternate hypothesis indication that difference between sample and control groups is due only to the chance. TRG is Target gene; REF is Reference gene. C.I. is confidence interval.

## Discussion

Discovering a suitable molecular marker to predict SR is a topic of current substantial research interest in andrology. In the first attempt between different azoospermia phenotypes, only SCOS was successfully correlated with *RBMY1* and *DAZ* genes, suggesting a significant positive association between these genes and successful SR^12^. The *BOll*/*GAPDH* mRNA ratio was assessed in different pathological phenotypes of azoospermia, and using a cut-off value of 0.5, sensitivity and specificity of 100% was achieved for SR^13^.

Technical improvements made the methodology of previous studies challenging, and therefore, the demand has risen for accurate and precise methods capable of diminishing biases. To address this urgency, RT-qPCR was introduced and applied in numerous recent studies. *ESX1* was the first reliable spermatogenesis molecular marker introduced with a significant (p = 0.04) concordance of 73.7%^14^. Additional testing of seminal fluid also confirmed the capacity of *ESX1* as a molecular marker of SR with 84% sensitivity, notwithstanding discrepancies between molecular and clinical outputs^15^. In a previous study, we improved the sensitivity of SR to 95.5% using *KDM3A* histone demethylase. However, we were unable to produce concordance between our molecular markers and pathological phenotypes^2^.

*TXNDC2* was correlated with SH phenotype in the present study, while *PRM1* and *PRM2* showed additional association with GCA/SCOS (Table 4). Notably, genome-wide integration of transcriptomics and antibody-based proteomics had previously determined that *TXNDC8* was a testis-specific protein as well, albeit as an extracellular equivalent of nuclear *TXNDC2*^11,16^. It seems logical to consider *TXNDC2* over *TXNDC8*, as protamine activation takes place in the nucleus. Furthermore, the association of *PRM2* but not *PRM1* with eMA was also notable. Specifically, these three genes could be altered at the very early stages of spermatogenesis, and when being expressed, could indicate the existence of germ cells. As we know, protamine activation occurs before they bind DNA, a potential role for thioredoxin. After the release of protamine precursors, a round of sequential phosphorylation and dephosphorylation strengthen protamines’ binding power to wrap around the corresponding DNA. A key event after dephosphorylation, completing the activation process, is the oxidation of protamine monomers to produce a head-to-tail dimer. Thioredoxins are oxidizing molecules acting on Cys residues, which are abundantly present in protamines. Therefore, synchronous downregulation of *TXNDC2* and *PRM1*/*PRM2* in SH and SCOS (the phenotypes of the most severe pathologies of sperm failure) could imply their importance for sperm production.

**Table 4:** mRNA expression and histological phenotypes.

Genes	ANOVA^a^		Multiple comparison between normal spermatogenesis and specimens with abnormal pathologies^b^			
	F	p	Case with differences	Mean	Std. errors	p
GAPDH	1.397	0.246				
RPL37	1.027	0.415				
TNP1	0.169	0.972				
TXNDC2	4.195	0.003	SH	− 5.593	1.416	0.017
PRM1	8.791	0.000	SH	− 0.275	0.046	0.000
			GCA\SCOS	− 0.234	0.051	0.003
PRM2	10.148	0.000	SH	− 0.284	0.046	0.000
			GCA\SCOS	− 0.252	0.050	0.001
			eMA	− 0.272	0.072	0.026

^a^A one-way between subjects ANOVA was conducted to compare the mRNA expression level of GAPDH, RPL37, TXNDC2, PRM1, PRM2 and TNP1genes in seminiferous hyalinization (SH), germ cell aplasia or Sertoli cell-only syndrome (GCA|SCOS), early maturation arrest (eMA), late maturation arrest (lMA), hypospermatogenesis (Hypo) and normal spermatogenesis (N). There was a significant main effect for pathology and TXNDC2, PRM1 and PRM2.

^b^Post hoc comparison using Scheffe test was done and all abnormal pathologies were compared with normal spermatogenesis. Pathologies with meaningful differences toward N were listed.

To future examine the observed synchronicity, a linear regression model was developed (Table 5). *TXNDC2* showed a strong correlation with *PRM1* (r = 0.761) and *PRM2* (r = 0.767). The coefficient of determination correlated up to 60% of *PRM1* and *PRM2* expression solely with *TXNDC2* expression. Moreover, *PRM1* was perfectly correlated (r = 0.993, p = 0.000) with *PRM2*, indicating that the value of *PRM2* could be anticipated from *PRM1* by 98.6%. Previous observations proposed similar correlations between two co-expressed protamines. Animal knockout models and our previous study confirmed *KDM3A*, itself under the control of *HIF1-a*, as the transcription factor of *PRM1* and *PRM2*^2,8,17^. It was also shown that the overexpression of thioredoxin could increase *HIF1-a* activity^18^.

**Table 5:** Linear regression analysis of target genes.

Genes of interest		Pearson correlation		ANOVA (p)	Coefficient of determination
Fixed factor	Dependent factor	R	p	F(p)	R^2^
TXNDC2	PRM1	0.761^a^	0.000	71.669 (0.000)	0.580^c^
TXNDC2	PRM2	0.767^a^	0.000	76.919 (0.000)	0.588^c^
PRM1	PRM2	0.993^b^	0.000	3467.630 (0.000)	0.986^c^

Several multiple linear regressions were calculated to predict the expression level of PRM1 and PRM2 based on TXNDC2 expression levels. A prediction was also made between PRM1 and PRM2. TXNDC2 significantly predicted PRM1, r = 0.761, p = 0.000. TXNDC2 also explained a significant proportion of variance in PRM1, R^2^ = 0.580, F = 71.669, p = 0.000. TXNDC2 significantly predicted PRM2, r = 0.767, p = 0.000. TXNDC2 also explained a significant proportion of variance in PRM2, R^2^ = 0.588, F = 76.919, p = 0.000. PRM1 significantly predicted PRM2, r = 0.993, p = 0.000. PRM1 also explained a significant proportion of variance in PRM2, R^2^ = 0.986, F = 3467.630, p = 0.000.

^a^A positive linear regression was found between TXNDC2 and both of PRM1 and PRM2.

^b^A strong positive linear regression was found between PRM1 and PRM2.

^c^Coefficient of Determination was shown positive values with strong predictability and with emphasizes on how well observed outcomes are replicated by the model.

**Figure 5:**
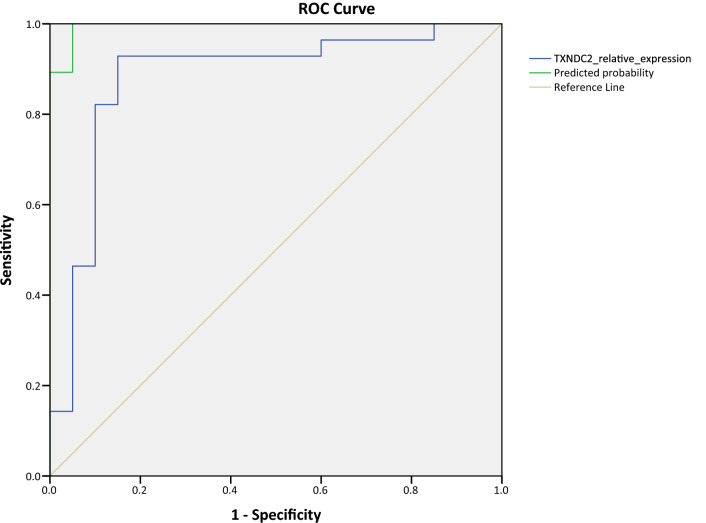
ROC curve analysis. TXNDC2 alone (Blue line) showed AUC = 0.880 significantly (p = 000,008). To assess the effects of protamines, logistic Regression model was built and, ROC curve analysis was performed. TXNDC2, PRM1, and PRM2 were all included in the regression model (green line). AUC value was significant and even more improved to 0.995 (SE = 0.0070, 95% CI 0.9816–1.000). The sensitivity and specificity were 95% (10% improvement) and 96.4% respectively.

Receiver operator characteristic (ROC) analysis was conducted to evaluate the predictive power of biomarkers. In the first step, the relative expression of *TXNDC2* was analyzed to understand its predictive potential regardless of SR. ROC curve analysis showed ROC value (AUC) = 0.880 for *TXNDC2* (Fig. 5, blue line). The recorded AUC value was statistically significant (p < 0.05). A sensitivity of 85% and specificity of 92.9% were determined for *TXNDC2*. To increase the diagnostic power of our potential biomarker, a logistic regression model of *TXNDC2* alongside *PRM1* and *PRM1*/*PRM2* was built based on the relative expression values. A regression model based on *TXNDC2* and *PRM1*, but not *PRM2*, showed an increased AUC value of 0.995 (p = 6.9279E−9). A 10% improvement in sensitivity was achieved at a cut-off value = 0. 2912 when *PRM1* and *PRM2* were introduced into the regression model (Fig. 5, green line). Therefore the improved sensitivity of 95% and specificity of 96.4% with the AUC value of 0.995 (SE = 0.0070, 95% CI 0.982–1.000) was revealed for the combined regression model of *TXNDC2*-*PRM1*-*PRM2*.

## Conclusions

*TXNDC2* was differentially expressed between positive and negative SR. Moreover, *TXNDC2* was correlated with phenotypes of severe azoospermia pathology (SH and SCOS). A strong correlation of *TXNDC2* with protamination genes was observed. ROC analysis applied to the multiple regression model demonstrated *TXNDC2*-*PRM1*-*PRM2* as robust molecular markers of SR with a sensitivity of 96.8% and specificity of 95.5%.

## Materials and methods

### Patients and samples

Azoospermic men were interviewed twice, before and after the operation. A sample was eliminated from analysis after the operation if the patient was unwilling to continue participating in the study. The mean age of the participating men was 30 ± 5 years old at the time of surgery. Inclusion criteria were men with primary idiopathic azoospermia who did not have any previous naturally born children. All the men were classified as having azoospermia by analyses of at least two semen samples, and they all suffered from a lack of sperm in the ejaculate. Men whom (i) had any chromosomal abnormality or (ii) *AZF* gene mutations, (iii) were severe smokers or addicted to drugs, (iv) had a history of testosterone therapy or (v) TESE or micro-TESE were excluded from this study. Approximately 50 mg of fresh testicular tissue was collected and submerged immediately into the RNAlater stabilizing reagent (Ambion Life Science, Austin, TX, USA, AM7024) according to the manufacturer’s instruction. The first piece of testicular tissue was used for RNA extraction, and the subsequent pieces for pathology and SR. Submerged samples were stored at 4 °C for 24 h and then processed for RNA extraction. A total number of 58 testicular tissue samples were collected entered this study. Nine of those samples were omitted as they presented with unknown pathology. According to the pathological results, out of the 50 samples included, 40 were diagnosed as non-obstructive and 10 as obstructive-control individuals. The exclusion criteria for samples were those with weak RNA integrity, variable Cqs even after multiple rounds of separate analyses, and without clear pathology.

### Ethics statement

Written informed consent was collected and a full explanation of the study was provided to azoospermic men before sampling. The experimentation and consent forms were approved by the institutional review board of the Isfahan University Ethical Committee. All procedures performed in the study involving human participants were in accordance with the 1964 Helsinki declaration and its later amendments or comparable ethical standards.

### SR technique

The Schlegel technique was employed and an expert surgeon performed all the micro-TESE open surgeries under a microscope to lessen the obstruction of testicular vessels^19^. Meticulous sperm processing with initial mechanical dissection of seminiferous tubules was followed by extensive exercise to ensure the maximum rate of retrieval^20^.

### Histological analysis

Hematoxylin and eosin (H&E) staining of paraffin-embedded tissues was performed according to the standard protocol^21^. A specialist pathologist examined two microscopic slides containing at least 100 different sections of seminiferous tubules for each specimen. The results were reported as follows: (i) N = normal spermatogenesis with all types of spermatogenic cell lineages in sections, (ii) SH = seminiferous tubule hyalinization, (iii) SCOS = Sertoli cell-only syndrome or germ cell aplasia, (iv) eMA = early maturation arrest, (v) lMA = late maturation arrest, (vi) Hypo = hypospermatogenesis. Individuals with normal spermatogenesis were considered to have obstructive azoospermia (OA), and these were the control individuals as per previous reports^15^. Other pathologies with abnormal spermatogenesis were classified as non-obstructive azoospermia (NOA).

### GEO meta-analysis

The GEO database was explored with the keyword “azoospermia” for microarray datasets. Rigid inclusion–exclusion criteria were applied as follows, and a total of nine datasets corresponding to Homo sapiens were found. Among these datasets, those including any treatments and therapies were excluded. Samples with the cryptorchidism phenotype and with detected mutations were also excluded. In this regard, GSE145467, GSE45885, GSE9194, GSE108886, GSE9210, GSE14310 were selected. All the candidate datasets were log_2_ scaled and quantile normalized if necessary. Hierarchical clustering of each dataset was illustrated using Euclidian distance. A principal component analysis (PCA) plot was drawn, and outliers were detected and removed. GSE9194 and GSE9210 were excluded due to low quality and low feature intersection with other datasets, respectively. SVA^22^ and Limma^23^ packages were used to remove batch effects, and subsequently, PCA and hierarchical clustering were used again to check the quality of the batch effect removal. The effect size of features was calculated using the Limma package with Benjamini–Hochberg correction. We applied p values to determine the corresponding false discovery rates (FDR). Finally, testis-specific thioredoxin gene 2 (*TXNDC2*) variation alongside protamination genes (*TNP1*, *PRM1*, *PRM2*) was recorded. Testis-specific thioredoxin gene 8 (*TXNDC8*) was not included in the GSE14310 dataset, and meta-analysis was performed on the resting GSE45885 and GSE108886 datasets. Software platform R 4.0.1 (R Foundation 3.6.2 for Statistical Computing, 2020, Austria) was used for meta-analysis.

### RNA isolation and cDNA synthesis

RNA extraction was carried out as described previously^2^. Nanodrop One (Thermo Scientific, USA) was used for quantification, and 1 μg of total RNA was treated with DNase I (Thermo Scientific, Lithuania; EN0522) according to the manufacturer’s instruction. TaKaRa PrimerScript II 1st strand cDNA synthesis kit (TaKaRa, Otsu, Japan; 6210B) was used to prime the first strand of cDNA randomly. Qualities of the extracted RNAs were confirmed by 2% conventional agarose gel electrophoresis stained with ethidium bromide (data not shown).

### Reverse transcription-quantitative real-time PCR (RT-qPCR)

Primers were adopted for RT-qPCR, and their concentration was optimized according to our previous study^2^. SYBR Premix Ex Taq II (TaKaRa; RR820L) was the quantifying dye in a Corbett 6000 Rotor-Gene thermocycler (Corbett Life Science, Mortlake, Australia). Equal amounts of cDNA were amplified in triplicate, and the values for the average cycle of quantification (Cq) were further analyzed.

### Melting curve analysis

After the final amplification, a melting curve analysis via green channel was performed according to the thermocycler manufacturer’s manual. The temperature was gradually increased (1.0 °C/s) from 65 to 95 °C, and the amount of emitted fluorescence was recorded continuously. The deviation of fluorescence change over temperature was plotted on the y-axis against the temperature on the x-axis using the Rotor-Gene embedded software v. 1.7.

### Gene expression analysis

*GAPDH* and *RPL37* were used simultaneously as reference genes for RT-qPCR data normalization based on our previous finding^2^. REST2009 (Qiagen, Germany) was used for statistical analyses.

### Statistical analyses

Raw mean Cqs were exported to SPSS v.21.0 (IBM Corp., Armonk, NY, USA), and normalization of the data was conducted if necessary. Normalized mean Cqs of the genes were compared between individuals with positive and negative SR using a t-test. A one-way between-subjects ANOVA-coupled with a Scheffe post hoc comparison was conducted to visualize the differences of mRNA expression levels between different testicular histopathologies. Multiple linear regression approaches were applied to model the relationship between the expression levels of *PRM1*, *PRM2*, and *TXNDC2*. A receiver operating characteristic curve (ROC) predictive model was obtained to demonstrate the predictive ability of the three expressed genes for SR. The area under the curve (AUC) was determined to assess the diagnostic accuracy. In all statistics, p values smaller than 0.05 were considered significant.

## Supplementary Information

Supplementary Information.

## Data Availability

The dataset (GSE145467, GSE45885, GSE9194, GSE108886, GSE9210, GSE14310) analyzed during the current study is available in the NCBI-Gene Expression Omnibus repository.
